# Comparisons of CVID and IgGSD: Referring Physicians, Autoimmune Conditions, Pneumovax Reactivity, Immunoglobulin Levels, Blood Lymphocyte Subsets, and HLA-A and -B Typing in 432 Adult Index Patients

**DOI:** 10.1155/2014/542706

**Published:** 2014-09-11

**Authors:** James C. Barton, Luigi F. Bertoli, J. Clayborn Barton

**Affiliations:** ^1^Department of Medicine, Brookwood Medical Center, Suite 626, 2022 Brookwood Medical Center Drive, Birmingham, AL 35209, USA; ^2^Southern Iron Disorders Center, Birmingham, AL 35209, USA; ^3^Department of Medicine, University of Alabama at Birmingham, Birmingham, AL 35294, USA; ^4^Brookwood Biomedical, Birmingham, AL 35209, USA

## Abstract

Common variable immunodeficiency (CVID) and immunoglobulin (Ig) G subclass deficiency (IgGSD) are heterogeneous disorders characterized by respiratory tract infections, selective Ig isotype deficiencies, and impaired antibody responses to polysaccharide antigens. Using univariable analyses, we compared observations in 34 CVID and 398 IgGSD adult index patients (81.9% women) referred to a hematology/oncology practice. Similarities included specialties of referring physicians, mean ages, proportions of women, reactivity to Pneumovax, median serum IgG3 and IgG4 levels, median blood CD56+/CD16+ lymphocyte levels, positivity for HLA-A and -B types, and frequencies of selected HLA-A, -B haplotypes. Dissimilarities included greater prevalence of autoimmune conditions, lower median IgG, IgA, and IgM, and lower median CD19+, CD3+/CD4+, and CD3+/CD8+ blood lymphocytes in CVID patients. Prevalence of Sjögren's syndrome and hypothyroidism was significantly greater in CVID patients. Combined subnormal IgG1/IgG3 occurred in 59% and 29% of CVID and IgGSD patients, respectively. Isolated subnormal IgG3 occurred in 121 IgGSD patients (88% women). Logistic regression on CVID (versus IgGSD) revealed a significant positive association with autoimmune conditions and significant negative associations with IgG1, IgG3, and IgA and CD56+/CD16+ lymphocyte levels, but the odds ratio was increased for autoimmune conditions alone (6.9 (95% CI 1.3, 35.5)).

## 1. Introduction 

Common variable immunodeficiency (CVID) and immunoglobulin (Ig) G subclass deficiency (IgGSD) are clinically and genetically heterogeneous disorders characterized by recurrent or severe infections of the upper and lower respiratory tract or other sites, selective deficiencies of Ig isotypes, and impaired antibody responses to common bacterial polysaccharide and protein antigens. Some adults with CVID also have decreased numbers or function of blood lymphocyte subsets, autoimmune conditions, or chronic inflammation [1–3]. Some adults with IgGSD have decreased numbers or function of blood lymphocyte subsets or autoimmune conditions [4–6]. Some human leukocyte antigen (HLA)-A and -B types and haplotypes (chromosome 6p) are associated with increased risk for CVID and IgGSD in adults [7–11]. HLA-A and -B haplotypes A*02, B*44 and A*03, B*07 were associated with transmission of both CVID and IgGSD immunophenotypes in some kinships [10].

Thus, we evaluated clinical and laboratory features of 432 consecutive white adult CVID and IgGSD index patients (34 CVID, 398 IgGSD) referred to a single practice because they had frequent or severe respiratory tract infections. We compared age at diagnosis, sex, specialties of referring physicians, autoimmune conditions, levels of serum Ig isotypes, blood lymphocyte subset levels, and HLA-A and -B types and haplotypes of CVID and IgGSD patients. Our results are discussed in the context of previous reports of clinical and genetic features of CVID and IgGSD.

## 2. Methods

### 2.1. Patient Selection

The performance of this work was approved by the Institutional Review Board of Brookwood Medical Center. All patients reported herein were referred to a hematology/medical oncology practice for further evaluation and management because they had (a) frequent or severe infections uncontrolled by antibiotic therapy and other management and (b) evidence of hypogammaglobulinemia.

We defined CVID in accordance with the criteria of the Pan-American Group for Immunodeficiency and the European Society for Immunodeficiency [12]. In adults, these criteria include serum IgG and IgA levels at least 2 standard deviations (SD) below respective means for age; absent isohemagglutinins or poor response to vaccines; and exclusion of other defined causes of hypogammaglobulinemia [12].

There is no generally accepted definition of IgGSD. One authoritative source defined IgGSD as serum levels of one or more IgG subclasses (IgG1-3) at least 2 SD below the mean(s) for age in the presence of normal serum IgG, with or without low serum IgA [13]. We accepted that case definition but also included patients with low IgG and normal IgA as having IgGSD. Thus, each of the present patients diagnosed herein to have IgGSD had primary immunodeficiency with non-protective serotype-specific serum IgG levels for or impaired responses to* Streptococcus pneumoniae* polysaccharide antigens.

We recommended that all patients accept vaccination with Pneumovax (Pneumovax 23, polyvalent pneumococcal vaccine; Merck, Sharpe & Dohme, Whitehouse Station, NJ) at diagnosis of primary Ig deficiency. We measured pre- and post-Pneumovax* Streptococcus pneumoniae* serotype-specific IgG antibodies, as possible. Postvaccination antibody panels were measured at least 4 weeks after the initial vaccination and before IgG replacement therapy was initiated.

We performed a computerized and manual search of charts of all white adults (≥18 years of age) in our practice who were referred as outpatients in the interval 1998–2013 because they had frequent or severe infections, typically of the upper and lower respiratory tract, and who were diagnosed to have CVID or IgGSD [10, 12, 13]. We designated the first persons in respective families diagnosed to have CVID or IgGSD as index patients. All patients resided in central Alabama.

We included observations on all index patients whose charts: (a) documented laboratory testing to establish their diagnosis of CVID or IgGSD, including flow cytometric analysis of blood lymphocyte subsets and HLA-A and -B typing and haplotyping and (b) contained a physician's recommendation that they be treated with either intravenous or subcutaneous IgG because they had frequent or severe infections. Autoimmune conditions were typically identified and characterized by referring physicians, our queries regarding autoimmune conditions, and medication reviews. Autoimmune conditions reported herein were diagnosed before diagnosis of CVID or IgGSD. We compiled histories of frequent or severe respiratory tract infections and autoimmune conditions in first-degree family members of each index patient.

### 2.2. Patient Exclusions

We excluded patients with (a) isolated subnormal IgA or IgM; (b) normal Ig levels with non-protective serotype-specific IgG levels for* Streptococcus pneumoniae* polysaccharide antigen(s); (c) hypogammaglobulinemia attributed to B-cell neoplasms, organ transplantation, immunosuppressive therapy, anticancer chemotherapy, or increased Ig loss; (d) polyclonal or monoclonal gammopathy; and (e) human immunodeficiency virus infection. We also excluded persons of African American descent because (a) certain HLA types and haplotypes differ significantly among Caucasians and African Americans in central Alabama [14–16]; (b) in adults, mean serum concentrations of Ig are often greater in persons of sub-Saharan African Native descent than in whites [17, 18]; (c) persons of sub-Saharan African Native descent occur infrequently in series of CVID or IgGSD patients [1]; and (d) such patients are also uncommon in our experience.

### 2.3. Laboratory Methods

Testing was performed at diagnosis of CVID or IgGSD before IgG replacement therapy was initiated. Serum IgG, IgG subclasses, IgA, and IgM were measured using standard automated methods. We defined mean ± 2SD as the normal or reference range for all Ig measurements, consistent with other investigators [10, 19–21]. Reference ranges are IgG 7.00–16.00 g/L; IgG1 4.22–12.92 g/L; IgG2 1.17–7.47 g/L; IgG3 0.41–1.29 g/L; IgG4 0.01–2.91 g/L; IgA 0.70–4.00 g/L; and IgM 0.40–2.30 g/L. Subnormal Ig levels were defined as those below the corresponding lower reference limit. Elevated serum Ig levels were defined as those greater than the upper reference limit. Antinuclear antibody titers of 1 : 80 or greater unexplained by other conditions were defined as positive.

IgG antibody test panels included measurements of antibodies specific for 6, 7, or 14* S. pneumoniae* serotypes, according to the year of diagnosis, physician choice, and insurance requirements. Six-serotype panels included these specificities: Types 1, 3, 14, 19, 23, and 51. Seven-serotype panels included these specificities: Types 4, 14, 19, 23, 26, 56, and 68. Fourteen-serotype panels included these specificities: Types 1, 3, 4, 8, 9, 12, 14, 19, 23, 26, 51, 56, 57, and 68. We defined serotype-specific IgG levels as either protective (≥1.3 mg/L) or nonprotective (<1.3 mg/L) [22]. Responses to vaccinations were defined as (1) increments in the number of protective antibody levels after vaccination (dichotomous variable) and (2) percentage increments of the numbers of protective antibody levels in the postvaccination panel compared to those in the prevaccination panel (continuous variable).

Blood levels of lymphocyte subsets were measured using flow cytometry. Reference ranges (mean ± 2SD) are CD19+ 12–645 cells/*μ*L; CD3+/CD4+ 359–1,519 cells/*μ*L; CD3+/CD8+ 109–897 cells/*μ*L; and CD56+/CD16+ 24–406 cells/*μ*L. Subnormal levels were defined as those below the corresponding lower reference limit. Elevated subset levels were defined as those greater than the upper reference limit.

HLA-A and -B alleles were detected using low-resolution DNA-based typing (polymerase chain reaction/sequence-specific oligonucleotide probe) in index patients [10]. HLA typing of family members of some index patients was performed to permit assignment of chromosome 6p haplotypes. In each index patient in whom a single A or B allele was detected by DNA-based typing, we verified the allele(s) and set phase to ascertain haplotypes of the index patient using HLA analyses of appropriate family members. Haplotypes were defined only by A and B alleles. We compiled all HLA-A and -B types in CVID and IgGSD patients. We also tabulated ten HLA-A and -B haplotypes that are associated with increased risk of CVID and IgGSD in central Alabama white index patients [7, 10].

### 2.4. Statistics

The final analytic data set consisted of observations on 432 index patients. Analyses were performed with Excel 2000 (Microsoft Corp., Redmond, WA) and GB-Stat (v. 10.0, 2003, Dynamic Microsystems, Inc., Silver Spring, MD). D'Agostino's test was used as a measure of normality. Descriptive data are displayed as enumerations, percentages, mean ± 1 SD, median (range), or mean (95% confidence intervals (CI)). Age at diagnosis data were normally distributed and were compared using Student's *t*-test (two-tailed). Because measures of some serum Ig isotypes, some blood lymphocyte subsets, and percentages of protective antibody levels were not normally distributed, we compared these data using the Mann-Whitney *U* test. Proportions were compared using Pearson's *χ* ^2^ test or Fisher's exact test, as appropriate. Linear correlations were performed using Pearson's technique. We performed logistic regressions on CVID (CVID and IgGSD as dichhltotomous variables) using all available independent variables except IgG and HLA-A and -B types. We computed the odds ratios of the significant variables and deviance of the final regression model. A value of *P* < 0.05 was defined as significant.

## 3. Results

### 3.1. General Characteristics of 432 Index Patients

There were 34 CVID patients and 398 IgGSD patients. The mean age at diagnosis of all patients was 49 ± 13 y. Mean ages of CVID and IgGSD patients did not differ significantly (data not shown). Women comprised 81.9% of the entire cohort. The proportions of women in CVID and IgGSD patients did not differ significantly (data not shown). Primary care, rheumatology, or otolaryngology specialists referred 79.4% of all patients. Percentages of referring medical specialists did not differ significantly between CVID and IgGSD patients (Table 1). Frequent or severe respiratory tract infections in first-degree family members were reported by 8 CVID patients (23.5%) and 153 IgGSD patients (38.4%) (*P* = 0.0843). Autoimmune conditions in first-degree family members were reported by 4 CVID patients (17.8%) and 82 IgGSD patients (20.6%) (*P* = 0.2154).

### 3.2. Autoimmune Conditions

The prevalence of autoimmune conditions was greater in CVID patients (55.9% versus 36.9%; *P* = 0.0292). In CVID and IgGSD patients with autoimmune conditions, 63.0% and 85.7% were women, respectively (*P* = 0.0045). Seven different autoimmune conditions were reported in CVID patients whereas 23 different autoimmune conditions were reported in IgGSD patients (Table 2). Sjögren's syndrome, Hashimoto's thyroiditis, and rheumatoid arthritis comprised 78.9% of autoimmune conditions in CVID patients and 39.4% of autoimmune conditions in IgGSD patients (*P* < 0.0001) (Table 2). The prevalence of Sjögren's syndrome and Hashimoto's thyroiditis was significantly greater in CVID patients (Table 2). Hypothyroidism due to Hashimoto's thyroiditis or unreported cause occurred in 44.1% of CVID patients and 15.8% of IgGSD patients (*P* < 0.0001) (Table 2).

### 3.3. *Streptococcus pneumoniae* Serotype-Specific IgG Antibodies

Panels of pre- and post-Pneumovax* S. pneumoniae *serotype-specific IgG antibodies were available in 28 CVID patients (82.5%) and in 315 IgGSD patients (79.1%) (*P* = 0.6572). No CVID patient had pre- and post-Pneumovax antibody test panels in which 100% of serotype antibodies were “protective.” In 26 IgGSD patients (8.3%), pre-and post-Pneumovax antibody panels included 100% protective levels of serotype antibodies (0 versus 8.3%, *P* = 0.1000). There was no increment in the number of protective IgG levels after Pneumovax in 7 CVID patients (25.0%) and in 114 IgGSD patients (36.2%) (*P* = 0.2350).

We also evaluated the post-Pneumovax percentage increments of the numbers of protective antibody levels in patients whose protective pre-Pneumovax antibodies were less than 100%. The median increment was 14.3% in 28 CVID patients and 14.3% in 289 IgGSD patients (*P* = 0.5382; Mann-Whitney *U* test).

We compared positivity for HLA-A*01, B*08 in CVID and IgGSD patients who had autoimmune conditions. This haplotype is part of a common, highly conserved, multigene and major histocompatibility complex (MHC)-linked ancestral haplotype associated with increased risk of CVID, subnormal IgA, and diverse autoimmune conditions [23]. Positivity rates for HLA-A*01, B*08 in CVID and IgGSD patients did not differ significantly (26.3% versus 18.4%, respectively; *P* = 0.4085).

### 3.4. Serum IgG, IgG Subclasses, IgA, and IgM

In CVID patients, distributions of IgG, IgG1, and IgG3 levels were normal. Other Ig levels were not normally distributed. In IgGSD patients, levels of all Ig isotypes were normally distributed. We determined correlations of IgG with the sum of IgG1–4 levels. In CVID patients, the Pearson correlation coefficient was 0.4766 (*P* < 0.0001). In IgGSD patients, the correlation coefficient was 0.7688 (*P* < 0.0001). In both CVID and IgGSD patients, the median IgG was slightly greater than the corresponding median of the sum of IgG1–4 (5.51 g/L versus 5.26 g/L, CVID; 7.58 g/L versus 7.49 g/L, IgGSD).

Median levels of all Ig isotypes were significantly lower in CVID patients except IgG3 and IgG4 which did not differ significantly between CVID and IgGSD patients (Table 3). By definition, IgG was subnormal in all CVID patients. IgG was also subnormal in 135 IgGSD patients (33.9%). The respective prevalence of subnormal IgG3 and subnormal IgG4 in CVID and IgGSD patients did not differ significantly. The combination of subnormal IgG1/IgG3 occurred in 58.8% of CVID patients and 28.9% of IgGSD patients (*P* = 0.0002).

Isolated subnormal IgG3 was detected in 121 of 398 IgGSD patients (30.4%) among whom the proportion of women (107/121; 88.4%) was higher than the proportion of women in IgGSD patients without isolated subnormal IgG3 (221/277; 79.8%) (*P* = 0.0371). No CVID patients and few IgGSD patients had elevated IgG. No patient had elevated IgG4 (Table 3).

By definition, all CVID patients had subnormal IgA and thus the median IgA level was lower in CVID patients than IgGSD patients. IgA levels were also subnormal in 5.2% of IgGSD patients. The median IgM level was significantly lower in CVID patients. The proportion of CVID patients who had subnormal IgM was also greater than that of IgGSD patients (Table 3).

### 3.5. Blood Lymphocytes

Levels of CD19+, CD3+/CD4+, CD3+/CD8+, and CD56+/CD16+ lymphocytes in both CVID and IgGSD patients were normally distributed, except CD3+/CD4+ lymphocytes in CVID patients. Median levels of CD19+, CD3+/CD4+, and CD3+/CD8+ lymphocytes were significantly lower in patients with CVID (Table 4). The proportions of CVID and IgGSD patients who had either subnormal or elevated CD19+ lymphocyte levels were low (Table 4).

Median levels of CD3+/CD4+ lymphocytes were significantly lower in CVID patients. Elevated CD3+/CD4+ lymphocyte levels occurred in 8.8% and 10.8% of CVID and IgGSD patients, respectively (Table 4). Median CD3+/CD8+ lymphocyte levels were significantly lower in CVID patients. Subnormal or elevated CD3+/CD8+ lymphocytes were uncommon in both CVID and IgGSD patients (Table 4). Median levels of CD56+/CD16+ lymphocytes did not differ significantly between CVID and IgGSD patients. Subnormal or elevated CD56+/CD16+ lymphocyte levels were uncommon in both CVID and IgGSD patients (Table 4).

### 3.6. HLA Types and Haplotypes

In CVID and IgGSD patients, positivity for HLA-A and -B types did not differ significantly (Tables 5 and 6). We also compared the frequencies of ten HLA-A and -B haplotypes selected because their respective frequencies were greater among Alabama CVID and IgGSD patients than in the general Alabama adult population [10]. In the present study, these haplotypes accounted for 47.1% of 68 chromosomes 6p in CVID patients and 37.6% of 796 chromosomes 6p in IgGSD patients (*P* = 0.1221). The respective haplotype frequencies in CVID and IgGSD patients did not differ significantly (Table 7).

### 3.7. Logistic Regressions on CVID

With initial regressions on CVID (versus IgGSD), we excluded many of the available independent variables because they were not significant. Among these exclusions was reactivity to Pneumovax as either a dichotomous or continuous variable. A final regression model on CVID revealed significant positive association with autoimmunity (*P* = 0.0247) and significant negative associations with serum IgG1, IgG3, and IgA (*P* < 0.0001, 0.0113, and <0.0001, resp.) and CD56+/CD16+ blood lymphocyte levels (*P* = 0.0141). The odds ratio of these significant associations was increased only for autoimmunity (6.9 (95% CI 1.3, 35.5)). This model accounted for 79% of the deviance in CVID occurrence.

## 4. Discussion

Similarities of 34 CVID and 398 IgGSD patients referred to a hematology/oncology practice determined using univariable analyses included specialties of referring physicians, mean ages at diagnosis, proportions of women, reactivity to Pneumovax, median serum IgG3 and IgG4 levels, median levels of CD56+/CD16+ blood lymphocytes, positivity for HLA-A and -B types, and frequencies of selected HLA-A, -B haplotypes. Dissimilarities included greater prevalence of autoimmune conditions, and lower median serum IgG, IgA, and IgM levels and lower median levels of CD19+, CD3+/CD4+, and CD3+/CD8+ blood lymphocytes in CVID patients.

Our observations differ somewhat from those in other CVID (or IgGSD) case series reports. All of the present patients were adults and all were referred due to frequent or severe infections alone, although 56% of the present patients had one or more autoimmune conditions at CVID/IgGSD diagnosis. In 334 European CVID patients followed for an average of 26 years, 71% had one or more inflammatory/autoimmune complications and the remainder had infections only [2]. In 476 US CVID patients, one or more of these complications occurred in 68% of the patients whereas infections were the only manifestation in the remaining patients [24]. At diagnosis of CVID in 303 European adults, some patients had splenomegaly (45%), lymphadenopathy (26%), autoimmune hemocytopenias (20%), or granulomatous disease (12%) [25]. In two large series of European CVID patients, enteropathy, autoimmunity, granulomatous disease, and splenomegaly were common and formed a set of interrelated features [25, 26]. Further, clinical phenotypes in CVID patients seem to be stable over long intervals [2, 24, 27]. Our informal observations suggest that enteropathy, granulomatous disease, and splenomegaly are uncommon in the present patients. Immune thrombocytopenia had not been diagnosed in any of the present patients before diagnosis of CVID or IgGSD, although this condition is the most prevalent autoimmune hemocytopenia in CVID patients [27–31].

There was a preponderance of women in both CVID and IgGSD patients in the present study, consistent with our previous report [10]. In studies of CVID, the proportions of men and women are typically equal [1, 12, 27]. In contrast, there is female predominance in adults with isolated subnormal IgG3 levels [4, 6, 32]. Likewise, the proportion of women among the present 121 IgGSD patients with isolated subnormal IgG3 was significantly greater than among IgGSD patients without isolated subnormal IgG3. Many of the present CVID and IgSD patients had autoimmune conditions. The prevalence of autoimmune conditions is widely acknowledged to be greater in women [33]. Regardless, these observations only partially explain the predominance of women in the present cohort.

Autoimmune conditions had been diagnosed in 56% of the present CVID patients before their referral. Analyzing follow-up observations of patients for the diagnosis of other autoimmune conditions was beyond the scope of the present work. In other CVID case series, 12–46% of patients had autoimmune conditions [2, 24, 34–36]. Autoimmune conditions occurred in 37% of the present IgGSD patients and in 13% of adults with IgGSD in Spain [5]. Although the diversity of autoimmune conditions in the present IgGSD patients was greater than that of CVID patients, no CVID patient and only one IgGSD patient had hemocytopenia attributed to autoimmunity. This result differs from that of other CVID case series reports [2, 24, 34–36]. Autoimmunity in CVID patients with the more common phenotype characterized by infections may also differ from that of patients who have the less common phenotype associated with polyclonal lymphocytic manifestations [24, 35]. Referral of almost one-quarter of the present patients by rheumatologists could account in part for the predominance of Sjögren's syndrome in both CVID and IgGSD patients. Hypothyroidism of all causes occurred in 44% and 16% of the present CVID and IgGSD patients, respectively. In two other reports, the prevalence of hypothyroidism in CVID patients was <1% [24] and 24% [37].

Median levels of CD19+ blood lymphocytes were significantly lower in CVID patients, but few had subnormal CD19+ blood lymphocytes, consistent with previous reports [27, 38]. Median blood levels of CD3+/CD4+ lymphocytes were also significantly lower in CVID patients. Subnormal CD3+/CD4+ blood lymphocyte levels occurred in a minority of the present CVID patients, consistent with previous reports [39–41].

T-lymphocytes of some CVID patients express surface marker patterns indicative of chronic activation [42]. In contrast to CD4+ T-lymphocytes, numbers of CD8+ T-lymphocytes of these patients may increase, explaining the inverted CD4/CD8 ratio reported in some patients with CVID [42]. CD8+ T-lymphocyte abnormalities have been associated with disturbed cytokine secretion [43], lower memory B-cell numbers and severe clinical courses [44], chronic or recurrent cytomegalovirus infections [45], and polyclonal expanded populations of large granular lymphocytes in association with splenomegaly [46]. Our informal observations suggest that CVID (or IgGSD) patients with these clinical attributes were uncommon in the present cohort, consistent with our observation that CVID patients had lower median blood CD3+/CD8+ lymphocyte levels and that no CVID patient had elevated blood CD3+/CD8+ lymphocyte levels.

Median blood levels of CD56+/CD16+ lymphocytes did not differ significantly between the present CVID and IgGSD patients. Subnormal or elevated CD56+/CD16+ levels were uncommon in both CVID and IgGSD patients. In contrast, Aspalter and colleagues reported that absolute and relative blood natural killer lymphocyte (CD16+/CD56+) numbers measured by flow cytometry in CVID patients were low [47].

HLA-A*01, B*08 is linked to CVID, subnormal IgA levels, and a putative deleterious gene(s) in the HLA class II region [7, 8, 11], presumably* IGAD1* [9, 48]. Thus, we had postulated that the frequency of haplotype A*01, B*08 would be greater in the present CVID patients (all of whom had subnormal IgA) than in IgGSD patients. Our postulate was not confirmed. In addition, proportions of the present CVID and IgGSD patients with autoimmune conditions who also had HLA-A*01, B*08 did not differ significantly. On the other hand, the respective frequencies of the ten selected haplotypes we studied were greater in patients with CVID or IgGSD than in the general Alabama adult population [10]. HLA-A and -B haplotypes A*02, B*44 and A*03, B*07 were associated with transmission of both CVID and IgGSD immunophenotypes in some kinships [10]. Thus, we infer that some of these haplotypes carry alleles that modulate blood Ig levels. Analysis of one family suggested linkage of CVID and subnormal IgA to chromosome 16q [49]. A genome-wide association study demonstrated linkage of these traits to numerous other loci [50].

CVID phenotypes have been associated with autosomal recessive mutations in genes that encode proteins important for B-cell function. These proteins include inducible costimulatory (ICOS) [51], CD19 [52], B-cell activating factor receptor [53], CD20 [54], CD21 [55], and CD81 [56]. Deleterious mutations in the corresponding genes have been identified in a small number of CVID patients, some from consanguineous kinships [27]. Mutations in* TACI* occur in approximately 10% of CVID patients [27], but their presence is not diagnostic of CVID [57]. IgG3 is the most polymorphic human IgG subclass and includes thirteen G3m allotypes (variants of Ig heavy G chains) which constitute six major G3m alleles of* IGHG3* on chromosome 14q32.33 [58]. Allotypes are characterized by differences in amino acid epitopes of the constant heavy G chains and are inherited in a Mendelian manner [58–61]. Thus, alleles that would account for subnormal IgG3 in some CVID and IgGSD patients may be linked to chromosome 14q32.33. MRL/*lpr* mice carrying a congenic H-2^b/b^ MHC interval exhibit several abnormalities including very low IgG3 [62]. A genome-wide association study of 366 CVID patients from four geographic areas confirmed a strong association of CVID with the MHC and demonstrated many significant associations with other regions [63].

There are uncertainties regarding the present results. CVID (and possibly IgGSD) patients who had lymphadenopathy, splenomegaly, enteropathy, or granulomatous disease may not have been referred to our practice or were excluded by our present selection criteria. The predominance of women in our cohort is incompletely explained. The reason that the variety of autoimmune conditions is less in the present CVID than IgGSD patients is unknown, although it is unlikely that erroneous reporting of autoimmune conditions would account for the differences we observed. We did not tabulate reports of autoimmune conditions that occurred in index patients after diagnosis of CVID or IgGSD and thus the present estimates of the occurrence of autoimmune conditions are conservative. Paired pre- and post-Pneumovax* S. pneumoniae* serotype-specific antibody evaluations were unavailable in 21% of the present patients. Evaluations for absent isohemagglutinins or poor response to vaccines are incomplete in other large CVID case series [25]. It seems unlikely that our conclusions about responsiveness to pneumococcal polysaccharide antigens would have changed greatly had additional observations been available for analysis. Flow cytometry analysis of subsets of CD19+, CD3+/CD4+, and CD3+/CD8+ blood lymphocytes and functional studies of lymphocytes may have demonstrated additional similarities or dissimilarities between CVID and IgSD patients. Performing extended HLA haplotyping, analyzing genes associated with CVID, and Gm allotyping were also beyond the scope of the present work.

## 5. Conclusions

Using univariable analyses, we demonstrated similarities and dissimilarities of CVID and IgGSD patients. Logistic regression on CVID (versus IgGSD) revealed significant positive association with autoimmune conditions and significant negative associations with IgG1, IgG3, IgA, and CD56+/CD16+ blood lymphocyte levels, but the odds ratio was increased only for autoimmune conditions (6.9 (95% CI 1.3, 35.5)).

## Figures and Tables

**Table 1:** Medical specialists who referred 432 adult CVID/IgGSD index patients^1^.

Referring physician	CVID (*n* = 34)	IgGSD (*n* = 398)	Value of *P*
Primary care, % (*n*)	29.4 (10)	27.4 (109)	0.7998
Rheumatology, % (*n*)	23.5 (8)	23.6 (94)	0.9907
Otolaryngology, % (*n*)	20.6 (7)	28.9 (115)	0.3018
Endocrinology, % (*n*)	8.8 (3)	3.0 (12)	0.1054
Gastroenterology, % (*n*)	5.9 (2)	1.5 (6)	0.1248
Pulmonology, % (*n*)	5.9 (2)	11.8 (47)	0.2309
Other^2^	5.9 (2)	3.8 (15)	0.3031

^1^CVID, common variable immunodeficiency; IgGSD, IgG subclass deficiency. Comparisons were made with Pearson's χ^2^ test or Fischer's exact test, as appropriate.

^2^Other: cardiology (2), gynecology (4), infectious disease (4), neurology (4), general surgery (2), and addictionology (1).

**Table 2:** Autoimmune conditions in 432 adult CVID/IgGSD index patients^1^.

Condition(s)	CVID (*n* = 34)	IgGSD (*n* = 398)	Value of *P*
Sjögren's syndrome, % (*n*)	20.6 (7)	6.5 (26)	0.0031
Hashimoto's thyroiditis, % (*n*)^2^	11.8 (4)	2.8 (11)	0.0059
Rheumatoid arthritis, % (*n*)	11.8 (4)	5.3 (21)	0.1199
Gastrointestinal tract, % (*n*)^3^	2.9 (1)	2.3 (9)	0.5634
Multiple sclerosis, % (*n*)	2.9 (1)	0.3 (1)	0.1514
Positive ANA, % (*n*)^4^	2.9 (1)	3.5 (14)	0.6664
Psoriatic arthritis, % (*n*)	2.9 (1)	1.3 (5)	0.3903
Autoimmune hearing loss, % (*n*)	0	0.3 (1)	0.9213
Autoimmune hemolytic anemia, % (*n*)^5^	0	1.0 (4)	0.7196
Diabetes mellitus, type 1, % (*n*)	0	1.3 (5)	0.6624
Graves' disease, % (*n*)	0	0.5 (2)	0.7820
Inflammatory arthritis, % (*n*)^6^	0	0.8 (3)	0.7815
Interstitial cystitis, % (*n*)	0	3.0 (12)	0.3690
Mixed connective tissue disorder, % (*n*)	0	1.3 (5)	0.6624
Myasthenia gravis, % (*n*)	0	1.0 (4)	0.7196
Polymyalgia rheumatica, % (*n*)	0	0.3 (1)	0.9213
Raynaud's phenomenon, % (*n*)	0	1.8 (7)	0.5610
Reiter's uveitis, % (*n*)	0	0.3 (1)	0.9213
Sarcoidosis, % (*n*)	0	0.3 (1)	0.9213
Skin and mucous membranes, % (*n*)^7^	0	2.3 (9)	0.4747
Systemic lupus erythematosus, % (*n*)	0	3.8 (15)	0.2863
Temporal arteritis, % (*n*)	0	0.5 (2)	0.7820
Thrombotic disorders, % (*n*)^8^	0	1.3 (5)	0.6624

^1^CVID, common variable immunodeficiency; IgGSD, IgG subclass deficiency. These autoimmune conditions were diagnosed before referral for CVID/IgGSD evaluation. Comparisons were made with Pearson's χ^2^ test or Fischer's exact test, as appropriate. Some patients had two or more autoimmune conditions.

^2^Hypothyroidism of unreported cause was reported in 11 CVID patients (32.4%) and 52 IgGSD patients (13.1%) (*P* = 0.0022).

^3^Crohn's disease (3), ulcerative colitis (3), and pernicious anemia (3).

^4^ANA, antinuclear antibody. Titers of 1 : 80 or greater unexplained by other conditions were defined as positive.

^5^Warm-reacting IgG autoantibody.

^6^Arthritis interpreted as autoimmune but not otherwise specified.

^7^Cutaneous psoriasis (3), erythema nodosum (2), vitiligo (2), alopecia areata (1), and Behcet's syndrome (1).

^8^Antiphospholipid syndrome (2), anticardiolipin antibody (1), and lupus anticoagulant (2).

**Table 3:** Serum immunoglobulins in 432 adult CVID/IgGSD index patients^1^.

Ig isotype	CVID (*n* = 34)	IgGSD (*n* = 398)	Value of *P*
Median IgG, g/L	5.51 (3.75, 6.92)	7.58 (3.43, 21.56)	<0.0001
Subnormal IgG, % (*n*)	100.0 (34)	33.9 (135)	<0.0001
Elevated IgG, % (*n*)	0	1.3 (5)	0.5109

Median IgG1, g/L	3.24 (2.42, 4.37)	4.10 (0.95, 14.60)	<0.0001
Subnormal IgG1, % (*n*)	97.1 (38)	56.3 (228)	<0.0001
Elevated IgG1, % (*n*)	0	0.3 (1)	0.7698

Median IgG2, g/L	1.34 (0.17, 3.50)	2.51 (0.31, 16.50)	<0.0001
Subnormal IgG2, % (*n*)	35.3 (12)	8.8 (35)	<0.0001
Elevated IgG2, % (*n*)	0	0.3 (1)	0.7698

Median IgG3, g/L	0.39 (0.07, 1.06)	0.33 (0.06, 1.76)	0.5821
Subnormal IgG3, % (*n*)	58.8 (20)	70.9 (282)	0.3336
Elevated IgG3, % (*n*)	0	1.5 (6)	0.4709

Median IgG4, g/L	0.08 (0, 0.29)	0.13 (0, 2.37)	0.0008
Subnormal IgG4, % (*n*)	5.9 (2)	3.3 (13)	0.4239
Elevated IgG4, % (*n*)	0	0	—

Median IgA, g/L	0.56 (0.04, 0.69)	1.55 (0.04, 5.55)	<0.0001
Subnormal IgA, % (*n*)	100.0 (34)	5.3 (21)	<0.0001
Elevated IgA, % (*n*)	0	2.5 (10)	0.3497

Median IgM, g/L	0.55 (0.10, 1.88)	1.00 (0.04, 5.16)	<0.0001
Subnormal IgM, % (*n*)	26.5 (9)	13.6 (54)	0.0407
Elevated IgM, % (*n*)	0	7.3 (29)	0.1032

^1^CVID, common variable immunodeficiency; IgGSD, IgG subclass deficiency; Ig, immunoglobulin; SD, standard deviation. Reference ranges (mean ± 2 SD) are IgG 7.00–16.00 g/L; IgG1 4.22–12.92 g/L; IgG2 1.17–7.47 g/L; IgG3 0.41–1.29 g/L; IgG4 0.01–2.91 g/L; IgA 0.70–4.00 g/L; and IgM 0.40–2.30 g/L. Subnormal Ig levels were defined as those below the corresponding lower reference limit. Elevated serum Ig levels were defined as those greater than the upper reference limit. Serum Ig levels are expressed as median (range). Comparisons were made with Mann-Whitney *U* test, Pearson's χ^2^ test, or Fisher's exact test, as appropriate.

**Table 4:** Blood lymphocytes in 432 adult CVID/IgGSD index patients^1^.

Lymphocyte subset	CVID (*n* = 34)	IgGSD (*n* = 398)	Value of *P*
Median CD19+ cells/*µ*L	172 (0, 631)	228 (5, 2340)	0.0051
Subnormal CD19+ cells, % (*n*)	5.9 (2)	0.5 (2)	0.0337
Elevated CD19+ cells, % (*n*)	0	2.8 (11)	0.4014

Median CD3+/CD4+ cells/*µ*L	783 (57, 1987)	937 (108, 2984)	0.0335
Subnormal CD3+/CD4+ cells, % (*n*)	14.7 (5)	3.8 (15)	0.0147
Elevated CD3+/CD4+ cells, % (*n*)	8.8 (3)	10.8 (43)	0.4988

Median CD3+/CD8+ cells/*µ*L	322 (110, 1304)	431 (48, 2106)	0.0453
Subnormal CD3+/CD8+ cells, % (*n*)	0	2.0 (8)	0.5170
Elevated CD3+/CD8+ cells, % (*n*)	0	7.8 (31)	0.0715

CD56+/CD16+ cells/*µ*L	118 (39, 295)	146 (4, 626)	0.1988
Subnormal CD56+/CD16+ cells, % (*n*)	0	3.5 (14)	0.3116
Elevated CD56+/CD16+ cells, % (*n*)	0	1.8 (7)	0.5618

^1^CVID, common variable immunodeficiency; IgGSD, IgG subclass deficiency; SD, standard deviation. Blood levels of lymphocyte subsets were measured using flow cytometry. Reference ranges (mean ± 2 SD) are CD19+ 12–645 cells/*µ*L; CD3+/CD4+ 359–1,519 cells/*µ*L; CD3+/CD8+ 109–897 cells/*µ*L; and CD56+/CD16+ 24–406 cells/*µ*L. Subnormal levels were defined as those below the corresponding lower reference limit. Elevated subset levels were defined as those greater than the upper reference limit. Blood lymphocyte levels are expressed as median (range). Comparisons were made with Mann-Whitney *U* test, Pearson's χ^2^ test, or Fisher's exact test, as appropriate.

**Table 5:** Positivity for HLA-A types in 432 adult CVID/IgGSD index patients^1^.

Type	CVID (*n* = 34)	IgGSD (*n* = 398)	Value of *P*
A∗01	38.2 (13)	31.4 (125)	0.4124
A∗02	52.9 (18)	51.1 (203)	0.8971
A∗03	26.5 (9)	25.1 (100)	0.8837
A∗11	2.9 (1)	9.6 (38)	0.1638
A∗23	2.9 (1)	3.8 (15)	0.6364
A∗24	11.8 (4)	15.8 (63)	0.0434
A∗25	5.9 (2)	3.8 (15)	0.3933
A∗26	5.9 (2)	4.8 (19)	0.5052
A∗28	2.9 (1)	0.8 (3)	0.2804
A∗29	2.9 (1)	8.8 (35)	0.2003
A∗30	0	4.8 (19)	0.2034
A∗31	5.9 (2)	4.3 (17)	0.4507
A∗32	8.8 (3)	6.0 (24)	0.5393
A∗33	0	2.0 (8)	0.5161
A∗34	0	0.5 (2)	0.8486
A∗66	0	0.8 (3)	0.7815

^1^HLA, human leukocyte antigen; CVID, common variable immunodeficiency; IgGSD, IgG subclass deficiency. Results are displayed as % (*n*). Comparisons were made with Pearson's χ^2^ test or Fischer's exact test, as appropriate.

**Table 6:** Positivity for HLA-B types in 432 adult CVID/IgGSD index patients^1^.

Type	CVID (*n* = 34)	IgGSD (*n* = 398)	Value of *P*
B∗07	29.4 (10)	22.9 (91)	0.3866
B∗08	38.2 (13)	25.4 (101)	0.1025
B∗13	0	4.3 (17)	0.2414
B∗14	2.9 (1)	9.0 (36)	0.1874
B∗15	11.8 (4)	14.1 (56)	0.4757
B∗18	8.8 (3)	7.3 (29)	0.4708
B∗27	2.9 (1)	6.8 (27)	0.3322
B∗35	8.8 (3)	14.1 (56)	0.2888
B∗37	2.9 (1)	2.3 (9)	0.5634
B∗38	8.8 (3)	2.8 (11)	0.0891
B∗39	0	3.5 (14)	0.1681
B∗40	17.7 (6)	8.8 (35)	0.0894
B∗41	0	1.0 (4)	0.7196
B∗44	44.1 (15)	30.2 (120)	0.0917
B∗45	0	2.3 (9)	0.4747
B∗47	0	1.0 (4)	0.7196
B∗48	0	0.3 (1)	0.9213
B∗49	0	3.8 (15)	0.2863
B∗50	0	2.8 (11)	0.3705
B∗51	14.7 (5)	8.3 (33)	0.1673
B∗52	0	2.5 (10)	0.4366
B∗53	2.9 (1)	1.3 (5)	0.3903
B∗55	0	3.5 (14)	0.3116
B∗56	0	1.3 (5)	0.6624
B∗57	2.9 (1)	7.0 (28)	0.3127
B∗58	0	0.5 (2)	0.8486
B∗60	0	1.3 (5)	0.6624
B∗62	2.9 (1)	0.5 (2)	0.2185

^1^HLA, human leukocyte antigen; CVID, common variable immunodeficiency; IgGSD, IgG subclass deficiency. Results are displayed as % (*n*). Comparisons were made with Pearson's *χ* ^2^ test or Fischer's exact test, as appropriate.

**Table 7:** Frequencies of HLA-A, -B haplotypes in 432 adult CVID/IgGSD index patients.

Haplotypes	CVID (*n* = 68 chromosomes)	IgGSD (*n* = 796 chromosomes)	Value of *P*
A∗01, B∗08	13.2 (9)	11.7 (93)	0.7034
A∗02, B∗44	16.2 (11)	9.9 (79)	0.1053
A∗02, B∗62	1.5 (1)	0.3 (2)	0.2182
A∗02, B∗60	0.0 (0)	0.1 (1)	0.9213
A∗03, B∗07	7.4 (5)	5.7 (45)	0.3569
A∗03, B∗14	0.0 (0)	1.6 (13)	0.3418
A∗03, B∗44	2.9 (2)	3.8 (30)	0.5314
A∗29, B∗44	1.5 (1)	3.4 (27)	0.3370
A∗31, B∗40	2.9 (2)	0.5 (4)	0.0745
A∗32, B∗14	1.5 (1)	0.6 (5)	0.3894

^1^HLA, human leukocyte antigen; CVID, common variable immunodeficiency; IgGSD, IgG subclass deficiency. These haplotypes were selected for study because their respective frequencies were significantly greater in Alabama CVID/IgGSD index patients than in population control subjects [10]. Results are displayed as % (*n*). Comparisons were made with Pearson's *χ* ^2^ test or Fischer's exact test, as appropriate. These haplotypes accounted for 47.1% of 68 chromosomes 6p in CVID patients and 37.6% of 796 chromosomes 6p in IgGSD patients (*P* = 0.1221).
